# A Federated Database for Obesity Research: An IMI-SOPHIA Study

**DOI:** 10.3390/life14020262

**Published:** 2024-02-16

**Authors:** Carl Delfin, Iulian Dragan, Dmitry Kuznetsov, Juan Fernandez Tajes, Femke Smit, Daniel E. Coral, Ali Farzaneh, André Haugg, Andreas Hungele, Anne Niknejad, Christopher Hall, Daan Jacobs, Diana Marek, Diane P. Fraser, Dorothee Thuillier, Fariba Ahmadizar, Florence Mehl, Francois Pattou, Frederic Burdet, Gareth Hawkes, Ilja C. W. Arts, Jordi Blanch, Johan Van Soest, José-Manuel Fernández-Real, Juergen Boehl, Katharina Fink, Marleen M. J. van Greevenbroek, Maryam Kavousi, Michiel Minten, Nicole Prinz, Niels Ipsen, Paul W. Franks, Rafael Ramos, Reinhard W. Holl, Scott Horban, Talita Duarte-Salles, Van Du T. Tran, Violeta Raverdy, Yenny Leal, Adam Lenart, Ewan Pearson, Thomas Sparsø, Giuseppe N. Giordano, Vassilios Ioannidis, Keng Soh, Timothy M. Frayling, Carel W. Le Roux, Mark Ibberson

**Affiliations:** 1 Novo Nordisk A/S, 2860 Søborg, Denmark; 2 Vital-IT Group, SIB Swiss Institute of Bioinformatics, CH-1015 Lausanne, Switzerland; 3 Genetic and Molecular Epidemiology Unit, Lund University Diabetes Centre, Department of Clinical Sciences, Clinical Research Centre (CRC), Lund University, Jan Waldenströmsgata 35, SE-20502 Malmö, Sweden; 4 Maastricht Center for Systems Biology, Faculty of Science and Engineering, Maastricht University, Paul Henri Spaaklaan 1, 6229 EN Maastricht, The Netherlands; 5 Department of Epidemiology, Erasmus MC, University Medical Center Rotterdam, 3000 CA Rotterdam, The Netherlands; 6 Global Biostatistics & Data Sciences, Boehringer Ingelheim Pharma GmbH & Co. KG, 88400 Biberach, Germany; 7 Institute of Epidemiology and Medical Biometry, CAQM, University of Ulm, 89081 Ulm, Germany; 8 German Center for Diabetes Research (DZD), 85764 Neuherberg, Germany; 9 Division of Population Health and Genomics, Ninewells Hospital and School of Medicine, University of Dundee, Dundee DD1 4HN, UK; 10 Nederlandse Obesitas Kliniek, Huis Ter Heide, 3712 BA Utrecht, The Netherlands; 11 University of Exeter Medical School, University of Exeter, Exeter EX1 2LU, UK; 12 Univ Lille, Inserm, CHU Lille, Pasteur Institute Lille, U1190 Translational Research for Diabetes, European Genomic Institute of Diabetes, 59000 Lille, France; dorothee.thuillier@region-academique-hdf.fr (D.T.);; 13 Data Science and Biostatistics Department, Julius Global Health, University Medical Center Utrecht, 3508 GA Utrecht, The Netherlands; 14 Fundació Institut Universitari per a la Recerca a l’Atenció Primària de Salut Jordi Gol i Gurina (IDIAPJGol), 08007 Barcelona, Spain; 15 ISV-Girona Research Group, Research Unit in Primary Care, Primary Care Services, Catalan Institute of Health (ICS), 08908 Barcelona, Spain; 16 Brightlands Institute for Smart Society (BISS), Faculty of Science and Engineering, Maastricht University, 6229 EN Maastricht, The Netherlands; 17 Department of Radiation Oncology (Maastro), GROW-School for Oncology and Reproduction, Maastricht University Medical Center, 6229 EN Maastricht, The Netherlands; 18 Nutrition, Eumetabolism and Health Group, Institut d’Investigació Biomèdica de Girona (IDIBGI-CERCA), Av. França 30, 17007 Girona, Spain; 19 Department of Medical Sciences, School of Medicine, University of Girona, 17003 Girona, Spain; 20 CIBER Fisiopatología de la Obesidad y Nutrición (CIBEROBN), Instituto de Salud Carlos III, 28029 Madrid, Spain; 21 Department of Diabetes, Endocrinology and Nutrition, Dr. Josep Trueta University Hospital, Av. França, s/n, 17007 Girona, Spain; 22 Department of Internal Medicine and CARIM School of Cardiovascular Diseases, Maastricht University, 6229 EN Maastricht, The Netherlands; 23 Department of Medical Informatics, Erasmus University Medical Center, 3000 CA Rotterdam, The Netherlands; 24 Research in Vascular Health Group, Institut d’Investigació Biomèdica de Girona (IDIBGI-CERCA), Parc Hospitalari Martí i Julià, Edifici M2, 17190 Salt, Spain; 25 Department of Genetic Medicine and Development, Faculty of Medicine, University of Geneva, 1 Rue Michel-Servet, CH-1211 Geneva, Switzerland; 26 Diabetes Complications Research Centre, University College Dublin, D04 V1W8 Dublin, Ireland

**Keywords:** federated database system, obesity, risk prediction, remote statistical analysis, bioinformatics

## Abstract

Obesity is considered by many as a lifestyle choice rather than a chronic progressive disease. The Innovative Medicines Initiative (IMI) SOPHIA (Stratification of Obesity Phenotypes to Optimize Future Obesity Therapy) project is part of a momentum shift aiming to provide better tools for the stratification of people with obesity according to disease risk and treatment response. One of the challenges to achieving these goals is that many clinical cohorts are siloed, limiting the potential of combined data for biomarker discovery. In SOPHIA, we have addressed this challenge by setting up a federated database building on open-source DataSHIELD technology. The database currently federates 16 cohorts that are accessible via a central gateway. The database is multi-modal, including research studies, clinical trials, and routine health data, and is accessed using the R statistical programming environment where statistical and machine learning analyses can be performed at a distance without any disclosure of patient-level data. We demonstrate the use of the database by providing a proof-of-concept analysis, performing a federated linear model of BMI and systolic blood pressure, pooling all data from 16 studies virtually without any analyst seeing individual patient-level data. This analysis provided similar point estimates compared to a meta-analysis of the 16 individual studies. Our approach provides a benchmark for reproducible, safe federated analyses across multiple study types provided by multiple stakeholders.

## 1. Introduction

Despite progress in communicating the challenges faced by patients living with obesity, the condition is considered by many as a lifestyle choice, a matter which can lead to disappointing treatment outcomes, with many patients regaining weight even after surgical or pharmaceutical interventions [1]. As a result, people with obesity tend not to seek medical solutions, clinicians tend not to treat them, and those paying for treatments tend not to reimburse them. The more recent view is that obesity is a serious, complex, progressive, chronic disease and that effective treatment strategies are required to improve the health outcomes for people with obesity [2]. Unfortunately, people with obesity are often viewed as all having the same disease without considering varying pathogenesis, risk profiles for complications, and treatment responses [3]. “Obesity”, even if not solely based on body mass index or body weight, is not a sufficient definition for many patients. The lack of understanding of disease pathogenesis and progression remains an obstacle.

The European Union-funded Innovative Medicines Initiative (IMI) project, Stratification of Obesity Phenotypes to Optimize Future Obesity Therapy (SOPHIA), is part of a momentum shift. The development of diagnostic tests using operational variables (routinely measured conventional metabolic, demographic, or novel parameters) may allow for more accurate predictions of the risk of obesity and response to obesity treatments [3]. High-quality data and analysis underpin the choice of these variables and their expected value.

The ability to leverage multiple datasets for biomarker discovery and patient stratification is a key component of many international research projects. However, for legal and ethical reasons, it is often not feasible to perform analysis by pooling individual-level data from the different studies together in a central repository. Whilst this is ultimately desirable from an analysis point of view, there exist alternatives to centralization for maximizing the value of multiple cohorts without having to embark on lengthy legal procedures for transferring data across different jurisdictions [4,5,6,7]. A classical approach is to perform meta-analyses, whereby individual studies are analyzed independently and the results are combined [7]. The disadvantage of such an approach is that it often involves sharing analysis scripts between several centers that themselves perform local analyses on their data. Because the data have been collected or measured in different ways and are often in different formats, this almost always requires adaptation of the analysis scripts to fit the local data, which is both time-consuming and error-prone. An alternative to this approach is federated analysis, where study data are stored on local servers which are set up and configured for remote analysis [8]. A prerequisite of such a system is that each dataset is formatted in the same way and that the same terms are used to describe the data. For example, demographics, measurements, and observations/outcomes are harmonized or mapped to standard or common dictionaries. Open-source tools have been developed that use the R statistical framework to perform remote analyses on multiple servers in parallel and integrate the results. In this system, cohort data are hosted on Opal database servers and analyzed using DataSHIELD R packages (https://www.datashield.org/, accessed on 15 August 2023) [9,10]. In SOPHIA, we chose to follow this route and set up a federated analysis system that currently comprises 16 harmonized obesity studies. Harmonization consists in mapping the original study variables to standardized dictionaries such as SNOMED [11] for describing clinical terms and LOINC [12], describing biomarker or other numeric measurements to enable interoperable cross-cohort analyses. The data are multi-modal, including those from research studies, routine health data, and data from industry-supported clinical trials. We opted to transform each study dataset to the Observational Medical Outcomes Partnership (OMOP) Common Data Model (CDM), following community guidelines for the FAIR (Findable, Accessible, Interoperable, Reusable) management of clinical data developed by the OHSDI community [13].

To facilitate the analysis of data, we have also built a suite of tools that work together with DataSHIELD, extending its capabilities to allow us to achieve the project’s goals [14]. The combined analysis of multiple cohorts through a federated database provides an exemplar of federated analytics of multiple data types and offers multiple ways to classify different obesity phenotypes, treatment responses, and disease trajectories. In this report, we present how we have set up the SOPHIA federated database and provide a simple proof-of-concept analysis illustrating how such a system can be used to generate results beyond what is possible from a more classical approach. We discuss the advantages, challenges, limitations, and opportunities of the system, how we are using it in SOPHIA, and how we plan to build on the system for the future.

## 2. Materials and Methods

### 2.1. Data Harmonization and Standardization

Data from studies were harmonized by mapping clinical, laboratory, demographics, medications, observations (e.g., from questionnaires), and other variables to common vocabularies SNOMED [11], LOINC [12], UCUM [15]**,** and RxNorm [16] following recommendations from the OHSDI community [17]. A minimum list of variables that were required for analysis was generated and used as a starting point to collect relevant data from each study. Mapping information for each study was stored in an Excel or CSV file so that the information could be parsed and extracted later. Once the mapping was complete for a cohort, the original data were transformed to OMOP CDM version 5.4 [18] in R, SAS, or SQL using the mapping information and CDM specifications. Each participating cohort performed the mapping of their original variables to the common vocabularies and the transformation to OMOP CDM. Both the harmonization to common vocabularies and the transformation to OMOP CDM were required to consolidate data from collaborative cohorts to make them interoperable and enable them to be analyzed together.

### 2.2. Set-Up and Deployment of Federated Nodes

To standardize the operating environment and facilitate the process of federated node set-up for partners, virtual machines (VMs) were prepared containing the complete set of software and the reference vocabularies required to run a federated node. Each VM contained the full Opal and DataSHIELD software stack (version 6.0, [19]). The VMs were installed in the local IT environment according to each node’s security policy. The two most popular hypervisor platforms, Oracle VirtualBox and VMWare, were chosen as the basis, and, for each platform, the two most popular operating systems, Ubuntu and CentOS, were selected to create VMs and then install on them the OPAL software stack. This gave the different collaborating nodes flexibility in selecting the VM suitable for their environment. The VM and the documentation to finalize the set-up of the federated nodes were made available. VMs’ set-up finalization (or “personalization”) consisted of changing the default passwords, adapting IP addresses to local network environment, and opening a dedicated port 8443 to/from a central proxy node hosted at the Swiss Institute of Bioinformatics (SIB) in Lausanne. Once a federated node was set up, harmonized data were loaded by local data administrators. Specifically, this process involved the following: (i) the creation of dedicated cohort databases (DBs) on a PosgreSQL database template pre-installed on the VM, one DB per cohort, and the inclusion of pre-loaded common vocabularies; (ii) loading data directly into the dedicated DB using the low-level “\COPY” command; and (iii) the activation of a set of standard OMOP foreign keys controlling the conformance of data loaded to the common vocabularies.

### 2.3. Proof-of-Concept Federated Analysis and Comparison to Meta-Analyses

OMOP-formatted data in 16 local PostgreSQL cohort databases were queried for gender, BMI, and systolic blood pressure on eight collaborating nodes using dsQueryLibrary [20], which implements standardized OMOP queries using Opal and DataSHIELD [9]. The remote data were evaluated and prepared for analysis using the dssPivot, dssSubset, dssRange, dssDeriveColumn, and dssScale functions from the R package dsSwissKnife [14,21]. A federated linear model was fitted for BMI against systolic blood pressure, correcting for gender on the 16 combined cohorts using ds.glm from the dsBaseClient R package [22]. Individual and pooled estimates were calculated using ds.glmSLMA (dsBaseClient). For pooled estimates random effect meta-analysis under maximum likelihood (ML) or restricted maximum likelihood (REML) was used. For comparison, a meta-analysis was also performed using ML and REML locally using estimates obtained from each cohort using *rma.uni* from the R package *metafor* [23]. Results were meta-analysed using both random and fixed effects models and a forest plot was generated using *ggplot2* in R [24].

## 3. Results

### 3.1. Identification of Cohorts for Federation

We started by drafting a list of potential clinical cohorts to make available for our federated analysis. To achieve this, we first collected a list of data types and variables that would be needed for a subsequent analysis and evaluated the cohorts based on the availability of these data. We also included more general criteria such as the number of individuals and follow-up time for the evaluation of the cohorts. Each cohort was contacted and invited to participate in the federated system explaining that patient-level data would be kept local and that access to the data for analysis was kept under the control of each participating cohort. Participation required that each cohort site set up and deploy two servers: a database server hosting an OMOP CDM-formatted database and an application server hosting software to enable remote querying and analysis. The specifications of the servers were defined based on the size of the data to be hosted and analyzed. Another prerequisite for participation was that the cohort data be harmonized and standardized to OMOP CDM. This required a data manager at each cohort site who intimately knew the cohort data and who could work together with the central SOPHIA team to prepare the data for inclusion into the federated database.

### 3.2. Data Standardization and Harmonization

Each participating cohort (Table 1) harmonized their data using common vocabularies and transformed their data to OMOP. This effort was coordinated through regular meetings and exchanges to address challenges, share experience, elaborate common approaches, and report progress. All results and decisions were kept on a common SharePoint including a set of files containing selections of vocabulary codes for several common entities present in the selected reference vocabularies. Such coordination is required because often a source term can be mapped to several almost-identical concepts, with different codes, within the vocabularies. For example, using the OMOP vocabulary mapping tool ATHENA [25], “aspirin” has 2208 hits (to concepts) in RxNorm, 1858 in SNOMED, and 65 in LOINC. It is, therefore, important that, for all concepts that will be used for a subsequent analysis across several cohorts, the same concept IDs are used for equivalent terms in the original data. As harmonization is an iterative process, repeated with each cohort, files were regularly updated to include new reference concept codes.

### 3.3. Federated Database Architecture

**Figure 1:**
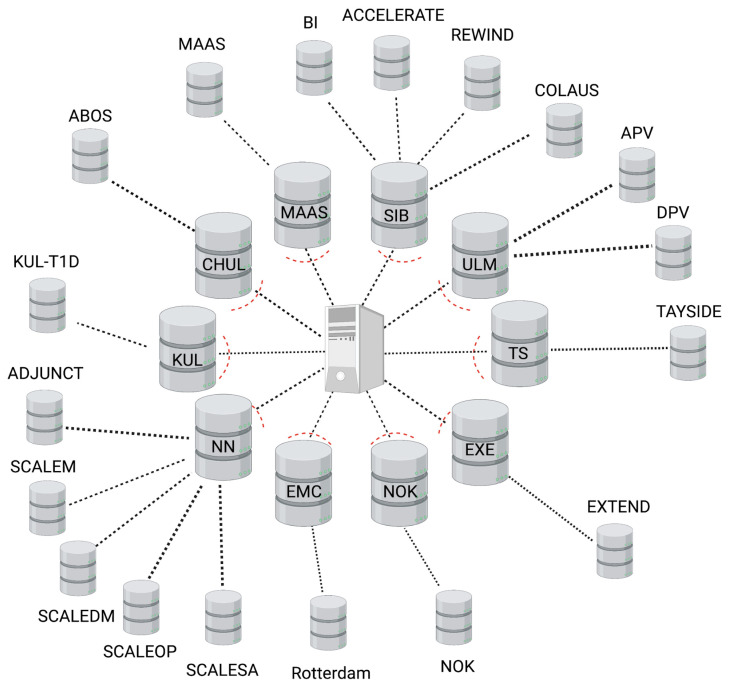
Schematic of the SOPHIA federated database showing a central node (proxy), 10 node servers (inner ring), and 18 harmonized cohort databases (outer ring).

The SOPHIA federated database system (FDB) consists of a central node server and a set of collaborating servers (see Figure 1). The central node acts as both an authentication server and a proxy, i.e., all communications (requests and replies) transit through the central node in a transparent way for the users. Every collaborating node consists of two servers: the front-end Opal server (opalsrv) and the back-end Opal database server (opaldb). The front-end server receives the user authentication status from the central node, handles the authorization of individual users (this optional step allows the nodes to increase controlled access on a user basis), and opens a working session in R connected to the federated system. The back-end Opal database server (opaldb) acts as a persistent storage of cohort data. The front-end receives the R requests, queries the back-end (opaldb), and returns the aggregated results. The original cohort data never leave the collaborating node. Opaldb responds to queries from opalsrv by sending only authorized data. Note that, when some collaborating nodes contain the data from several cohorts, all are loaded onto the same host opaldb server.

It was the responsibility of each cohort to set up, maintain, and administer each node, such as, for example, installing a firewall to monitor and control incoming and outgoing network traffic or validating software updates which are deployed centrally. The node-based architecture is scalable and versatile. Indeed, the set-up of multiple nodes can be performed in parallel. In addition, the federated system continues to function correctly even when nodes are added or removed.

### 3.4. Federated Database Access

As the federated database contains sensitive data, a secure access policy is critical to safeguard against unauthorized access. The set-up is configured so that access is controlled in two phases: there is first a user authentication phase, which is followed by a local authorization step. User authentication (verification of a user’s identity against a centrally managed list of users) is managed using Agate (https://www.obiba.org/pages/products/agate/, accessed on 15 August 2023), a dedicated software provided with the Opal suite by OBIBA [39]. In this first step, each collaborating node checks the validity of the user’s identity using a dedicated authentication request to Agate. The subsequent authorization phase checks that the user is entitled to access the data node he or she requested. This phase proceeds separately on each of the collaborative nodes engaged by the initial user request. The username is checked against a whitelist of authorized users, and, if present, the user receives an access token for the node. Access does not mean that the user can view or download individual-level data, but it means that the user can execute functions using the R DataSHIELD and dsSwissKnife packages, which can only return summary-level data. It is also important to note that neither the central administrator nor the remote users have direct access (e.g., by SSH) to collaborative servers, nor do remote users have direct access to the central server.

A typical user session through R proceeds as follows: a user initiates a session by sending a list of desired federated nodes to connect to, along with their own username and password; the software stack orchestrates communication with each of the collaborating nodes via the central proxy server; each of the federated nodes engaged by the request checks the user authentication claim against the authentication server Agate; a session creation request is passed to each node’s whitelist; and, if authorized, the user can work remotely on the engaged node. An analysis session lasts for a certain period but expires after a time-out configured at each local node.

### 3.5. Federated Proof-of-Concept (PoC) Analysis

**Figure 2:**
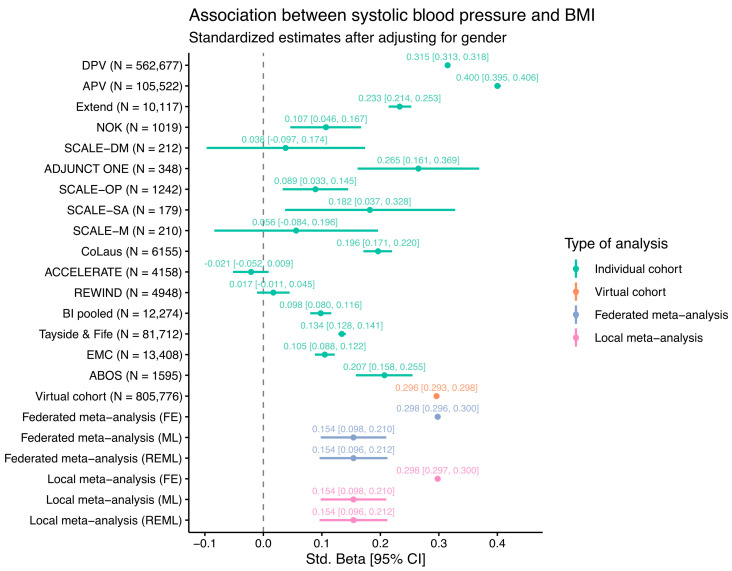
Estimates of linear regression modelling of BMI ~ SBP + Gender from individual federated cohorts, a “virtual” or federated cohort, and meta-analyzed cohorts using fixed-effect (FE), maximum likelihood (ML), or restricted maximum likelihood (REML) estimations (federated and local). The 95% confidence intervals are shown as horizontal lines and within square brackets.

As an initial test of how the SOPHIA federated database works, a PoC analysis was carried out, performing a linear regression comparing two commonly available variables: BMI ~ Systolic blood pressure (SBP) + Gender. After omitting rows with missing values, the regression analysis was performed in three ways: (i) individually, reporting estimates from individual cohorts; (ii) meta-analyzed, reporting results from two commonly used approaches for combining estimates from separate cohorts, remotely and locally; and (iii) federated, where an iterative glm algorithm [5] was used to obtain a pooled estimate using data from all cohorts. The meta-analyses in (ii) were carried out using both a fixed-effect (FE) approach, in which cohorts were assumed to share a single common effect with all the variance in the observed estimates being attributable to within-study sampling errors, and two variants of a random-effect approach (maximum likelihood, i.e., ML, and restricted maximum likelihood estimation, i.e., REML), in which the estimates were allowed to vary between cohorts, with this variance stemming from both within and between cohorts. The iterative glm used in (iii) did not allow estimates to vary between cohorts. The results are shown in the forest plot in Figure 2.

The estimates for individual cohorts are mostly positive, as would be expected, except for the ACCELERATE and REWIND clinical trials, where no association was evident. This is likely because these clinical trials enrolled only CVD patients with the majority on medication for high blood pressure, potentially disrupting any underlying association between BMI and SBP. The other cohorts show a wide range of estimates, illustrating differences between the study populations. Many smaller studies such as the SCALE or ADJUNCT ONE clinical trials have wide 95% confidence intervals due to the relatively low number of individuals in these studies. For the virtual cohort (N = 805,776), where the data from all individual cohorts were combined into a single regression model, the estimate is approximately the same as those of the fixed-effect federated and local meta-analyses. The wide range of estimates also suggests that the fixed-effect assumption is unlikely to hold, thus leading to notably different estimates for the models using random-effect methods (ML and REML). Overall, the results from the meta-analysis methods implemented in the federated database system are almost identical to the results obtained via traditional meta-analysis methods.

It is important to note that the goal of this PoC analysis was not to seek new insights into the data but rather to demonstrate the power of combining data from multiple cohorts in a federated analysis. Indeed, given that many diverse studies with different populations ranging from clinical trials to patient registries are included here, the estimates from the virtual cohort and the meta-analysis should not be interpreted in a medical context. In the SOPHIA federated database, users are free to choose which cohorts to include for analysis and can subset patients according to all available data, thus enabling a large amount of flexibility.

## 4. Discussion

We present a federated database system of multiple obesity studies that has been developed as part of the IMI SOPHIA project. This system provides a template for federated analysis approaches that has many advantages and some disadvantages over current solutions, which we discuss below. The primary goal of the database is to aid the discovery of biomarkers for the stratification of people with obesity by leveraging data from multiple diverse clinical datasets. The system we have put in place enables federated analysis without the disclosure of any sensitive data, thus bridging data silos and avoiding the need for data transfer agreements.

The task of setting up such a database requires the engagement of many different actors including data experts, clinical data managers, medical experts, and IT personnel. One critical element for success is the harmonization and standardization of the different cohorts enabling their interoperability within the same system. This task is complicated as it requires in-depth knowledge of the cohort data in terms of data content and structure, knowledge of common vocabularies for standardization, and detailed medical knowledge to correctly associate clinical events or outcomes to standardized medical dictionary terms. We used a common standard, OMOP CDM [13], an open-source community data model which is designed for observational clinical studies. OMOP, which has become increasingly popular in recent years [40,41,42,43,44,45,46], ensures the interoperability of data by providing both a relational database model and a large set of standardized concepts derived from common vocabularies covering a range of different data types, such as demographics, measurements, observations, and medications. As part of the process of harmonization, specific concept terms need to be selected that best match the term in the source dataset being harmonized. This poses several challenges as there are often many equivalent terms from the same or different vocabularies. Another challenge is that there are often multiple ways in which patient data can be correctly encoded in OMOP. For example, if a study individual had a particular medical condition (e.g., cardiovascular disease) in the past, this can be assigned to a single concept such as “history of cardiovascular disease” or to two concepts, one generic concept “history of” and a second disease concept “cardiovascular disease”. Ideally, all collaborating nodes should format their data in the same way so that the same queries can be performed on each node. In order to coordinate mapping activities, we set up regular meetings (every 2 weeks) between data experts working on different cohorts, where progress was assessed and any mapping issues were discussed. Documents detailing the mapping of individual cohorts were shared on a common drive so that each data expert could consult the mapping from other cohorts. This way of working was effective for the mapping of the first few cohorts, but, as the number of cohorts grew, it became more difficult to track the mapped variables in all cohorts. In the future, using a system such as CaRROT-Mapper [47], which is designed for tracking and the harmonized mapping and transformation of multiple cohorts to OMOP, could be advantageous.

The SOPHIA federated database hosts data on 10 IT servers (the nodes) in different locations across Europe that comprise the main federated database infrastructure. The deployment and configuration of these node servers were performed in parallel to the data harmonization and transformation described above. A node server is made up of two parts, a database server for hosting the data and a computer server for performing the federated analysis. The set-up of these servers was performed by local IT groups in close collaboration with the SIB, who was responsible for the overall architecture and user access. The process was initiated by the provision of virtual machines containing the necessary database and software packages for local installation, but many technical discussions were required with local IT personnel to tailor the installations and configuration to the local IT policies and security requirements. For example, in some instances, the node was deployed and configured on a private cloud (AWS or Azure) or within a safe haven, a demilitarized zone (DMZ), or a trusted research environment (TRE). In all these deployments, specific firewall rules needed to be configured to enable the node server to communicate with the central proxy server for user authentication. Since cyber security is of the utmost importance, it was critical to work together with the local teams to make sure that there was no risk of data breach or cyber-attack through the local node.

The SOPHIA federated database is built on open-source software: Opal for data storage and DataSHIELD for analysis, provided by the open-source software for the epidemiology OBIBA community [9,10]. DataSHIELD is a privacy-preserving data analysis software framework that allows researchers to analyze sensitive data stored across different locations without physically moving the data to a central repository. It works together with the Opal software for secure data storage, organization, and management allowing researchers to upload, store, and manage datasets while ensuring data privacy and security.

The DataSHIELD framework allows statistical analysis to be performed on individual-level data stored securely at each participating site. Instead of combining the data, DataSHIELD uses distributed computing to perform computations across separate datasets while keeping individual-level data safe. Importantly, pooled analysis is possible within this system even though individual-level data are never shared or brought together even for an instant. For more technical details, interested readers are referred to the original publication describing the DataSHIELD concept [5]. Using this system, only the aggregated results are shared, and no identifiable data are exposed to other sites or researchers. DataSHIELD has been successfully used in an increasing number of studies [48,49,50,51,52,53,54] and provides a range of statistical analysis methods that can be remotely executed on federated nodes. This system enables the analysis to be brought to the data rather than having to copy the data to an analysis server, which can be difficult or impossible due to legal or ethical restrictions which often differ between countries and jurisdictions. DataSHIELD is agnostic of data structure and, in principle, does not require remote data to be formatted or standardized. However, being able to query multiple data sources without having to deal with different formats and naming conventions is essential for the usability of such a system. As described above, in SOPHIA, we chose to format all cohort data to OMOP CDM, which meant that we needed to develop new software tools to be able to query data remotely from the OMOP databases using DataSHIELD. To this end, we developed an R package, dsQueryLibrary, that implements standardized OMOP queries in DataSHIELD, thus expanding its usability to OMOP-formatted data. This is particularly relevant in the context of projects such as IMI EHDEN, which is in the process of formatting data from over 180 partners in 29 different countries across Europe to this community standard [55]. Since the databases will already exist once this project is completed, enabling federated computing on the ensemble of these OMOP datasets could be achieved by deploying application servers running software similar to those we have deployed in SOPHIA.

We present a proof-of-concept analysis demonstrating the use of the SOPHIA federated database by performing a simple linear regression of BMI against systolic blood pressure (SBP) across 16 cohorts. Our aim was not to provide new biological findings or insights into the data but rather to demonstrate the similarities and differences between a federated analysis, an analysis run on individual cohorts, and a standard meta-analysis. Whilst, in this example, most results show the expected positive association between BMI and SBP, the effect sizes are very different, which is most likely due to sample size differences between cohorts. As expected, the federated analysis and the (fixed-effect) meta-analysis showed very similar point estimates, thus confirming the validity of the federated cross-cohort analysis using the system. We are in the process of developing federated software for more complex algorithms using this system which will hope will reveal new insights into the combined studies.

Within SOPHIA, our analysis efforts are centered around the stratification of patients according to the risk of disease complications, such as cardiovascular disease (CVD) and chronic kidney disease (CKD). In order to achieve this, we are implementing methodologies into the federated system that will allow unsupervised and supervised patient clustering. Methods such as UMAP, Similarity Network Fusion, RCCA, and Common Dimensions (ComDim) are being implemented to complement PCA, k-means clustering, and other methods available in the dsSwissKnife R package [14]. These tools will enable such analyses to be performed on multiple cohorts simultaneously and, where possible, offer the possibility to run analyses on virtual cohorts as demonstrated in the PoC example.

Whilst the federated system enables a powerful analysis which was previously not possible, there are a few limitations. First, and probably most important, performing the same or similar analyses across different cohorts necessitates that the cohorts in question have the same or equivalent measurements or patient characteristics to make the cross-cohort analysis meaningful. Within SOPHIA, there are many diverse studies that have been designed to answer different clinical and research questions. Whilst harmonization and standardization are essential, they are only part of the solution, and significant efforts are needed at the analysis stage to select the most appropriate cohorts to perform a particular analysis. This is because the fine details of exactly which data are available across cohorts can be interrogated only once cohorts have been standardized and connected. Another issue frequently encountered is that data are often collected at varying levels of granularity in different cohorts. This means that, even when standardized, additional harmonization is often required by the analyst to decide on the equivalence of terms between cohorts. A simple example of this might be whether to treat a plasma glucose measurement the same as a blood glucose measurement or whether medication information based on a questionnaire is equivalent to that based on prescription in the context of a particular analysis.

In contrast to traditional analyses, which are often performed on a single server, a federated analysis performs calculations on multiple servers in parallel. The performance speed is, therefore, dependent on the computing power and capacity of each federated node, the size of the data, and the complexity of the analysis. For large cohorts, merely loading the data into memory can be time-consuming and subsequent analysis cumbersome if the corresponding server is not adequately sized or powered. In order to circumvent potential problems at the analysis stage, it is preferable to host cohort data on a scalable server, e.g., a private cloud, so that node capacity can be scaled according to the analysis requirements.

Despite these limitations, federated systems like the one we have implemented in SOPHIA offer the possibility to access and analyze multiple large datasets in real time without compromising data security. The more datasets are included into the federated database, the more powerful it will become, and the system is highly scalable since any new datasets can be added as long as they are harmonized and hosted on a server that can be connected into the federated database. The system we are using for the SOPHIA federated database (DataSHIELD) is one of several technologies for federated analysis; others include federated learning, swarm learning, ledger-based systems such as blockchain, as well as improved encryption or storage methods such homomorphic encryption and data clean rooms [6,56,57,58]. These technologies promise to revolutionize the way in which we perform research using sensitive data, helping us to maximize the return on the investment of past and future clinical studies. However, each technology has advantages and disadvantages depending on the data being analyzed and the scientific question being addressed. In the future, one can imagine a system of harmonized databases that can be accessed through a central platform offering different flavors of federated analysis depending on the analysis requirement. For example, currently, for deep learning using multiple distributed medical imaging resources, one might use swarm learning, while, for building statistical models from diverse data, DataSHIELD is a good option, and, for methods where the pooling of data is essential, encryption and blockchain technologies can ensure that data can be transferred and analyzed securely and removed once analysis has been completed.

## Figures and Tables

**Table 1:** Cohorts and datasets available in the SOPHIA federated database. [key: study design: PROS = prospective cohort; RET = retrospective cohort; OBS = observational, CROS = cross-sectional; and RCT = randomized control trial. Available data: CL = clinical; GO = genomics; TO = transcriptomics; PO = proteomics; MO = metabolomics; and MCO = microbiomics; Bold indicates that the data are currently available for analysis through the federated database].

Cohort	Study Design	Individuals	Data Types
ABOS [26]	OBS/Bariatric surgery	1602	**CL**,GO,**MO**,**PO**,MCO
ACCELERATE [27]	Placebo arm of RCT	6047	**CL**
ADJUNCT-ONE [28]	Placebo arm of RCT	348	**CL**
APV Registry [29]	OBS	126,947	**CL**
BI pooled trials ^1^	Placebo arm of RCT	13,125	**CL**
CoLAUS [30]	PROS	6733	**CL**,GO,TO,MO
DPV Registry [31]	PROS, OBS	638,031	**CL**
EXTEND [32]	OBS, CROS	10,134	**CL**,GO
KUL-T1D	RET	1400	CL
Maastricht Study [33] ^2^	PROS	3451	CL,GO,MO
NOK Discovery [34]	CROS	564	**CL**,GO
REWIND [35]	Placebo arm of RCT	4949	**CL**
Rotterdam Study [36]	PROS	14,926	**CL**,GO,TO,PO,MO,MCO
SCALE Diabetes [37]	Placebo arm of RCT	212	**CL**
SCALE Maintenance [37]	Placebo arm of RCT	210	**CL**
SCALE Obesity and Prediabetes [37]	Placebo arm of RCT	1242	**CL**
SCALE Sleep apnea [37]	Placebo arm of RCT	179	**CL**
Tayside/Fife T1D &T2D [38]	OBS	87,050	**CL**
Total		912,299	

^1^ patients randomized to placebo or active comparator—patients randomized to the BI drug were excluded—in the following trials: T1DM (1245.69, 1245.72) and T2DM (1218.18, 1218.20, 1218.22, 1218.63, 1218.66, 1218.74, 1218.89, 1245.19, 1245.20, 1245.23, 1245.25, 1245.31, 1245.33, 1245.36, and 1245.49). ^2^ Cohort not yet online in the SOPHIA federated database.

## Data Availability

No new data were created or analyzed in this study. Data sharing is not applicable to this article.
